# Prevalence of mental health problems in Chinese schoolchildren: The influence of measuring impact score and combining information from multiple informants

**DOI:** 10.1186/s13034-020-00346-2

**Published:** 2020-11-09

**Authors:** Li Liu, Shuang Li, Wen Pan, Lijuan Wang, Yang Zheng, Xiaoxia An, Yan Zhou, Yanxia Li, Jun Na, Rui Zhang, Huijuan Mu, Wen Dong, Yuan Gao, Wei Sun, Guowei Pan, Lingjun Yan

**Affiliations:** 1 Institute of Preventive Medicine, China Medical University, Shenyang, 110122 P. R. China; 2 Institute of Chronic Diseases, Liaoning Provincial Center for Disease Control and Prevention, No. 77 Puhe Road, Shenyang North New Area, Shenyang, Liaoning P. R. China; 3 Anshan Municipal Center for Disease Control and Prevention, Anshan, P. R. China; 4 Panjin Municipal Center for Disease Control and Prevention, Panjin, P. R. China; 5 Benxi Municipal Center for Disease Control and Prevention, Benxi, P. R. China; 6 Dandong Municipal Center for Disease Control and Prevention, Dandong, P. R. China; 7 Department of Personal Resource, Dalian Medical College, Dalian, P. R. China; 8 Research Center for Universal Health, School of Public Health, China Medical University, Shenyang, 110122 P. R. China

**Keywords:** Prevalence, Mental health problems, Multiple informants, Impact score, Combined information

## Abstract

**Background:**

To measure the effects of using different combinations of multiple informants and the impact score on the estimated prevalence of mental health problems in Chinese schoolchildren.

**Methods:**

Complete information on the Strengths and Difficulties Questionnaire (SDQ) were obtained from students (S), parents (P), and teachers (T) for 4986 schoolchildren (11–17 years-old). We used 3 criteria to determine the prevalence of mental health problems: SDQ cut-off value (previously established in the United Kingdom), SDQ cut-off value plus an impact score of 1 or more, or plus an impact score of 2 or more. A student was defined as having a mental health problem if any informant (S, P, or T) classified the child as ‘abnormal’. We compared the prevalence of mental health problems determined from 1 informant, 2 informants, and 3 informants.

**Results:**

The prevalence of overall mental health problems increased with rising number of informants, but decreased with increasing impact score. When the impact score was not considered, the prevalence was 8.2% to 14.2% when rated by 1 informant, 18.8% to 24.7% when rated by 2 informants, and 28.3% when rated by all 3 informants. Failure to measure the impact score led to a two to threefold greater estimate of the prevalence of mental health problems.

**Conclusions:**

The types, number, and combinations of multiple informants and use of the impact score must be considered when comparing the results of different studies. It is preferable to use multiple informants and have the impact score taken the impact into account to reflect the real burden of mental health burden in children and adolescent.

## Background

Many children have psychological problems, and 10–20% of children and adolescents worldwide have mental health problems [1, 2]. Early detection and treatment of mental health problems is crucial, because if these troubles are undetected and untreated, they can increase in severity with age, and lead to chronic, complex, disabling, and expensive complications in adulthood [3–5]. However, the assessment of mental health problems in children and adolescents can be difficult, because diverse methodological approaches are used. Children have different exposures to risk factors and protective factors, and the cultural context in which mental health problems occur may alter estimates of their prevalence, which range from 1.81% to 39.4% according to previous studies [1, 2, 6]. Current screening methods rely on the expression of certain symptoms or impairments in everyday functioning to identify at-risk individuals who need further evaluation and potential treatment [7].

Although many instruments are available to screen children with mental health problems, the Strengths & Difficulties Questionnaire (SDQ) has become one of the most-used instruments because it is relatively short, user-friendly, has good validity in different cultures, and has more positive wording than other common questionnaires [8]. The SDQ uses a multi-informant approach, and is suitable for studies of populations in the general community, in which most children are healthy. The use of SDQ results from multiple informants is valuable, due to the situational nature of psychosocial problems [9–11]. In the absence of a gold standard measure for assessing mental health problems in children and youths, a multi-informant multimodal approach is considered best [12–16].

Previous studies reported significant differences in the prevalence of mental health problems when children and adolescents were rated by different informants or different combinations of informants [17]. Good mental health is more than simply the lack of symptoms or low levels of symptoms, but is also the ability to function adaptively within important life domains [18]. Psychiatric symptoms lead to distress and functional impairment, and assessment of the impact of symptoms on the lives of children is central to clinical practice, and a key indicator of the need for mental health care [19]. An impact supplement of the SDQ enables informants to report on possible burden and distress [20]. When distress or impairment criteria are used for diagnosis, the prevalence of mental health problems is significantly lower [17, 21]. The DSM-IV and ICD-10 coding implicitly assume that impact is sufficiently separable from symptoms, and can be considered individually. This suggests that distress and impairment resulting from symptoms should be adequately measurable on their own, and should add predictive value to a diagnosis based on symptoms alone [21].

Although use of multiple informants and impact score are crucial for making valid assessments of the prevalence of psychological problems in children and adolescents, most studies have used the SDQ to screen for mental health problems using only one informant, and have not considered the impact score. There is little research on the influence of considering the impact score on the estimated prevalence of mental health problems in children [17]. For example, 9 previous studies used the SDQ to evaluate the prevalence of mental health problems in Chinese schoolchildren using 1 informant [22–30]; eight of these studies used a parental version of the SDQ, and the rates ranged from 8.2 to 19.3%, and 1 study used a student version of the SDQ, and the rate was 10.7%. However, none of these 9 studies considered the impact score. Another previous study used 3 informants to assess the prevalence of emotional problems in Chinese schoolchildren [31], and reported that the prevalence rated by parents (8.2%), students (8.3%), and teachers (8.5%) were similar. However, the prevalence based on different combinations of 3 informants has not yet been determined. Therefore, the true prevalence of mental health problems in Chinese schoolchildren, based on different individual informants, different combinations of informants, and impact scores, remains to be established.

The purpose of the present study is to measure the influence of different combinations of multiple informants and the impact score on the estimated prevalence of mental health problems in Chinese schoolchildren.

## Methods

### Sample

The specifics of the sampling procedure were previously described [32]. This study was a cross-sectional survey of Chinese schoolchildren who were 6–17 years-old, and attended primary, junior, or senior schools. These children lived in 3 cities and 3 rural counties of Liaoning Province, in northeastern China. A 2-stage sampling procedure was conducted: schools were randomly selected, and a random sample of students was selected from each school. After obtaining written consent from parents, an informant-rated version of the SDQ was completed by the parents (P), teachers (T), and students (S; 11–17 years-old). The study protocol was reviewed and approved by the Institutional Review Board of the Liaoning Provincial Center for Disease Control and Prevention.

There were 9806 students eligible for the study, 9298 students returned the forms, and qualified SDQs were available for 8488 students. This included 8055 P-SDQs from parents (94.90%), 8418T-SDQs from teachers (99.18%), and 5446 S-SDQs from 5451 students aged 11–17 (99.91%). For this study, students were only included if SDQs from all 3 sources were available for students aged 11–17. By these criteria, 4986 students (91.5%) had complete data, and 465 students (8.5%) had incomplete data. The mean age of the included students was 13.9 years (SD: 1.9 years), and boys made up 48.7% of the sample.

### Instruments

The SDQ is an instrument used to screen for mental health problems that asks about the occurrence of 25 behaviors in the past 6 months (0 = not true, 1 = somewhat true, 2 = certainly true) in the following dimensions: emotional problems, conduct problems, hyperactivity/inattention, peer relationship problems, and prosocial behavior. The items in the first 4 subscales generate a “total difficulties score” (range 0–40). The SDQ impact score is generated by the sum of 5 items: 1 item about distress and 4 items about social impairment (in family life, friendships, learning activities, and leisure activities) [20]. The Chinese version of the SDQ has been validated [33].

### Statistical analysis

Descriptive statistics (means and standard deviations) for the SDQ subscales, total difficulties score, and impact score, are presented. A paired t-test was used to determine the significance of differences in SDQ total score and sub-scores from the parent, teacher, and student. The corresponding values for the schoolchildren aged 11–17 from Shanghai [33] and UK [34] were also presented and compared. Because Chinese norms for these scales are not yet available, we used the SDQ cut-off points of ‘abnormal’ from a previous study in the United Kingdom to define students with mental health problems [34]. To investigate the role of symptoms versus impact in assessment of mental health problems, we combined information from two parts of the multiple informant-reported SDQ (symptoms and impact) by identification of children with abnormal symptom scores and impairment (impact score ≥ 1 or impact score ≥ 2).

To determine differences in the prevalence of mental health problems assessed by different individual informants and different combinations of informants, we calculated the rates by 1 informant (S, P, or T), 2 informants (S + P, S + T, or P + T) and all 3 informants (S + P + T). When combining 2 or 3 informants, we defined the student as ‘positive’ if any one classified the child as ‘abnormal’, according to the corresponding cut-off values [34]. We only analyzed the prevalence of overall mental health problems and not specific problems, in this paper.

## Results

We initially recruited 5451 schoolchildren who were 11–17 years-old (Table 1). A total of 49.1% were male, 52.7% were under 15 years-old, 66.1% were from single-child families, and 7.3% had divorced parents. The percentages of completed SDQs were 93.5% for parents, 98.0% for teachers, and 100.0% for students. A total of 4986 students (91.5%) had completed SDQs from all 3 three informants, and we further analyzed these students below.

**Table 1:** General information of 5451 schoolchildren aged 11–17

	N	%
Gender		
Male	2678	49.13
Female	2773	50.87
Age		
11–14	2872	52.69
15–17	2579	47.31
Single child (yes/no)	3604	66.12
Mather education		
Primary	1201	22.03
Junior	2343	42.98
Senior	1294	23.74
College	613	11.25
Parental divorced (yes/no)	399	7.32
SDQ informants		
Self	5449	99.96
Parent	5095	93.47
Teacher	5342	98.00
Number of informants		
1	2	0.04
2	463	8.49
3	4986	91.47
All	5451	100.00

The data of 4986 children who were assessed by all 3 informants were used for subsequent analyses

Table 2 shows that the 3 individual informants had significantly different mean scores in all SDQ domains except for hyperactivity in the total SDQ score, and in the impact score. The students’ scores were the highest in total problems, impact, emotional symptoms, and prosocial behavior, followed by parents and then teachers. Teacher scores were the highest for peer problems, followed by parents and then students. Parent scores were the lowest for conduct problems.

**Table 2:** Paired t-test of the means of SDQ scores for 4986 schoolchildren with complete information rated by three informants

	Student			Parent			Teacher		
	Liaoning	Shanghai [33]	UK [34]	Liaoning	Shanghai [33]	UK [34]	Liaoning	Shanghai [33]	UK [34]
Emotional symptoms	2.52 ± 2.24	2.3	2.8	1.88 ± 1.91	1.76	1.83	1.66 ± 1.83	1.81	1.75
Conduct problems	2.63 ± 1.40	2.16	2.2	1.31 ± 1.30	1.53	1.5	1.44 ± 1.63	1.47	1.68
Hyperactivity	*3.18 ± 2.16*	3.32	3.8	*3.21 ± 2.21*	3.77	2.3	*3.18 ± 2.43*	3.63	2.72
Peer problems	2.64 ± 1.56	2.85	1.5	2.77 ± 1.60	2.72	1.62	2.90 ± 1.64	2.67	1.78
Prosocial behavior	7.70 ± 1.83	7.32	8	7.50 ± 1.94	7.13	2.07	6.94 ± 2.39	6.99	2.55
Total problem	10.96 ± 5.04	10.6	10.3	*9.16 ± 4.77*	9.77	5	*9.17 ± 5.58*	9.58	5.77
Impact score	0.52 ± 1.22	–	0.2	*0.26 ± 0.83*	–	0.4	*0.25 ± 0.80*	–	0.4

–: no data

For each row, italic values indicate no significant difference based on a t-test; all other values are significantly different

The general pattern of our SDQ scores for Liaoning schoolchildren is similar to that previously reported for schoolchildren aged 11–17 years-old from Shanghai [33] and the UK [34] (Table 2). However, the student impact score in our study (0.52) was much greater than the parent impact score (0.26) and teacher impact score (0.25); in contrast, the student impact score in the UK study (0.2) was much less than the parent impact score (0.4) and teacher impact score (0.4). Impact scores were not available for the study in Shanghai.

Table 3 compares the prevalence of overall mental health problems defined by different criteria: (a) abnormal symptoms using different combinations of 3 informants; (b) abnormal symptoms using different combinations of 3 informants and an impact score of 1 or more; and (c) abnormal symptoms using different combinations of 3 informants and an impact score of 2 or more. In all cases, we used the UK cut off values for abnormal symptoms and impairment [34]. The rate of mental health problems increased significantly with increasing number of informants, but these numbers were much smaller when we also considered impact score. More specifically, when we considered 1 informant, the prevalence of mental health problems was greater when rated by the students themselves (5.9% to 14.2%) than by teachers (3.3% to 14.0%) or parents (2.5% to 8.2%). When we considered 2 informants, the prevalence was greater when rated by students + teachers (8.7% to 24.7%), than students + parents (7.5% to 18.8%) or parents + teachers (5.5% to 19.8%). The prevalence was greatest when we considered all 3 informants together (10.1% to 28.3%). When we included impact scores in the assessment of all 3 informants, the prevalence of overall mental health problems was 10.1% (impact score ≥ 2) and 14.5% (impact score ≥ 1), much lower than when we only considered symptom scores alone (28.3%).

**Table 3:** Comparison of mental health problems by different combinations of abnormal symptoms and impact scores with UK cut off value for SDQ and impact scores [34]

	P		T		S		P + T		S + P		S + T		SPT	
	N	%	N	%	N	%	N	%	N	%	N	%	N	%
Abnormal symptoms	407	8.16	699	14.02	706	14.16	989	19.84	937	18.79	1231	24.69	1412	28.32
Abnormal + Impact ≥ 1	176	3.53	254	5.09	437	8.76	397	7.96	542	10.87	635	12.74	723	14.50
Abnormal + Impact ≥ 2	123	2.47	166	3.33	294	5.90	274	5.50	375	7.52	433	8.68	505	10.13

Abnormal symptoms were defined by an SDQ cut-off value previously established for children in the UK [34]

*P* parent, *T* teacher, *S* student, *P + T* parent and teacher, *S + P* student and parent, *S + T* student and teacher, *SPT* student and parent and teacher

## Discussion

Consistent with previous findings in Shanghai [33] and the UK [34], we found that the 3 individual informants had significant differences in SDQ scores in most domains. The low to moderate correlations among the 3 informants for total score (0.219 to 0.443) and impact score (0.101 to 0.296) confirms previous findings that the 3 informants are inconsistent in their assessment of mental health problems in children and adolescents [8, 35, 36]. The students reported significantly higher average scores than adults and teachers in most SDQ domains, except for peer problems and hyperactivity problems. This is consistent with the interpretation that schoolchildren are more cognitively or psychologically aware of subjective symptoms and internal burdens, and that adults are more aware of observable or objective phenomenon [37]. In general, when considering all 3 informants, we found similar mean SDQ subscale scores in Chinese and British children and adolescents (Table 2). However, comparisons of the impact scores of Chinese and British parents (0.26 *vs.* 0.40) and Chinese and British teachers (0.25 *vs.* 0.40) indicated significantly lower scores for the Chinese [34]. In contrast, Chinese children assigned significantly higher impact scores than British children (0.52 vs*.* 0.20). This may suggest that Chinese adults tend to undervalue the impact of such problems, or that the impairment is less obvious to observers in China, possibly due to a difference in the knowledge or attitude of adults to mental health problems of schoolchildren in these countries.

In general, we observed a similar prevalence of overall mental health problems (8.2%-14.2%) based on a previously established SDQ cut-off score [34] from individual informants without considering impact. This agrees with previous studies of Chinese schoolchildren using the SDQ scores from 1 informant (8.2–19.3%) [22–30]. As expected, we observed a significantly greater prevalence of mental problems as the number of informants increased, and a decreased prevalence when considering the impact score. The prevalence of mental health problems was greater when rated by students (5.9% to 14.2%) than parents (2.5–8.2%) or teachers (3.3–14.0%). In addition, consideration of 2 informants indicated the prevalence was greatest for students + teachers, followed by students + parents, and then teachers + parents. This confirms the presence of a big difference in perceiving and rating children’s behavior and mental health problems between students and observers (parents and teachers) [13]. These results also suggest that schoolchildren themselves have an important role in assessing the prevalence of overall mental health problems [21, 38].

However, a previous study reported that self-reported information from students provided poorer screening than parents or teachers, the combinations of parent + teacher and teacher + student provided better screening than parent + student, and parent + teacher + student provided the most sensitive screening [10]. Although there is no ‘gold standard’ about which informants to use to assess mental health problems in schoolchildren, our results support the view that multiple informants provide better information than any individual informant. In addition, the large variations between the students and observers, as well as their combinations, suggests that the type and number informants must be considered when comparing the prevalence of mental health problems among studies.

Similar to the results of the BELLA study [17], we found that the prevalence of overall mental health problems rated by individual informants and different combinations of informants declined substantially when additional impairment criteria are considered. For example, the prevalence assessed by all 3 informants was 28.3% when impact score was not considered, but was 14.5% for an impact score of 1 or more and 10.1% for an impact score of 2 or more. The prevalence of 28.3% represents children who met the symptom criteria alone, but included those who were not necessarily impaired by the symptoms; this may provide a greater sensitivity, but may also overestimate the mental burden, and lead to false positive diagnoses and unnecessary additional screening and treatment [10]. We believe the prevalence based on an abnormal SDQ score plus an impact score of 1 or more (14.5%) could reflect ‘real mental health problems’, because this combination considers symptoms and perceived functional damage. Interestingly, the prevalence determined by three informants that considers SDQ score and an impact score of 2 or more (10.1%) was very close to the overall prevalence of DSM-IV disorders (9.49%) assessed using the Developmental and Well-Being Assessment (DAWBA) in the same population [32]. This combination reflects a higher diagnostic quality (discrimination between respondents with and without a psychiatric diagnosis) when screening for a psychiatric disorder than the symptom scales of the SDQ [10, 17].

The present results should be viewed in the context of several limitations. First, this was a study of schoolchildren for whom full information was available from all 3 informants. Children who left school or could not get information from their parents or teachers were excluded. This may have led to an underestimate of the prevalence of mental health problems, because such children have greater risk of mental health problems in developed and developing countries. Second, we used the criteria and cutoff values from the UK, and there were big differences in the impact scores between Chinese and British children and adults. However, the purpose of the present study was to assess the influence of using multiple informants and impact scores on the prevalence of mental health problems in Chinese schoolchildren. Although it would be preferable if regional norms were available, we believe the internal comparisons among different subgroups, defined by different combinations of informants and impact scores, are comparable and valid. Third, because each teacher must rate all the students in a class (about 40), the teacher may not have sufficient time to carefully assess each child. It is unclear whether this is related to the lower average SDQ scores from teachers than from students and parents.

## Conclusions

The results of the present paper confirm the presence of substantial differences in the prevalence of mental health problems in Chinese schoolchildren when rated by different informants, different combinations of informants, and when impairment scores are considered. The assessment of mental health problems using the SDQ depends on the purpose of the survey or screening, the types, numbers, and combinations of informants, the cut-off values, and the use of impact score [10]. It is preferable to use multiple informants and consider impact score to better assess the actual burden of mental health problems in children and adolescents.

## Data Availability

The datasets generated and analysed during the current study are not publicly available because of our agreement with the parents of schoolchildren, but are available from the corresponding author on reasonable request.

## Abbreviations

SDQ: Strengths & Difficulties Questionnaires
DSM-IV: Diagnostic and Statistical Manual of Mental Disorders
ICD-10: International Classification of Diseases 10th Revision

## References

[1] Kieling C, Baker-Henningham H, Belfer M, Conti G, Ertem I, Omigbodun O (2011). Child and adolescent mental health worldwide: evidence for action. Lancet..

[2] Kato N, Yanagawa T, Fujiwara T, Morawska A (2015). Prevalence of Children's Mental Health problems and the effectiveness of population-level family interventions. J Epidemiol.

[3] Bricker D, Davis MS, Squires J (2004). Mental health screening in young children. Infants Young Child.

[4] Patel V, Flisher AJ, Hetrick S, McGorry P (2007). Mental health of young people: a global public health challenge. Lancet.

[5] Parks RJ, Kalberg RJ, Carter EW (2007). Systematic screening at the middle school level: score reliability and validity of the student risk screening scale. J Emot Behav Disord.

[6] Canino G, Alegría M (2008). Psychiatric diagnosis-is it universal or relative to culture?. J Child Psychol Psyc.

[7] Levitt JM, Saka N, Romanelli LH, Hoagwood K (2007). Early identification of mental health problems in schools: the status of instrumentation. J School Psychol.

[8] Stone LL, Otten R, Engels RCME, Vermulst AA, Janssens JMAM (2010). Psychometric properties of the parent and teacher versions of the strengths and difficulties questionnaire for 4- to 12-year-olds: a review. Clin Child Fam Psychol Rev.

[9] Achenbach TM, McConaughy SH, Howell CT (1987). Child/adolescent behavioral and emotional problems: implications of cross-informant correlations for situational specificity. Psychol Bull.

[10] Goodman R, Ford T, Simmons H, Gatward R, Meltzer H (2000). Using the Strengths and Difficulties Questionnaire (SDQ) to screen for child psychiatric disorders in a community sample. Brit J Psychiat.

[11] Goodman R, Renfrew D, Mullick M (2000). Predicting type of psychiatric disorder from Strengths and Difficulties Questionnaire (SDQ) scores in child mental health clinics in London and Dhaka. Eur Child Adoles Psy.

[12] De Los RA, Kazdin AE (2004). Measuring informant discrepancies in clinical child research. Psychol Assess.

[13] De Los RA, Kazdin AE (2005). Informant discrepancies in the assessment of childhood psychopathology: a critical review, theoretical framework, and recommendations for further study. Psychol Bull.

[14] Verhulst FC, Dekker MC, Van der Ende J (1997). Parent, teacher and self-reports as predictors of signs of disturbance in adolescents: whose information carries the most weight?. Acta Psychiat Scand.

[15] Achenbach TM (2005). Advancing assessment of children and adolescents: commentary on evidence-based assessment of child and adolescent disorders. J Clin Child Adolesc Psychol.

[16] Mash EJ, Hunsley J (2005). Evidence-based assessment of child and adolescent disorders: issues and challenges. J Clin Child Adolesc.

[17] Ravens-Sieberer U, Wille N, Erhart M, Bettge S, Wittchen HU, Rothenberger A (2008). Prevalence of mental health problems among children and adolescents in Germany: results of the BELLA study within the National Health Interview and Examination Survey. Eur Child Adoles Psy.

[18] Rapee RM, Bogels SM, Van der Sluis CM, Michelle GC, Ollendick T (2012). Annual research review: conceptualizing functional impairment in children and adolescents. J Child Psychol Psyc.

[19] Dang HM, Weiss B, Trung LT (2016). Functional impairment and mental health functioning among Vietnamese children. Soc Psych Psych Epid.

[20] Goodman R (1999). The extended version of the Strengths and Difficulties Questionnaire as a guide to child psychiatric caseness and consequent burden. J Child Psychol Psyc.

[21] Stringaris A, Goodman R (2013). The value of measuring impact alongside symptoms in children and adolescents: a longitudinal assessment in a community sample. J Abnorm Child Psychol.

[22] Wang JN, Liu L, Wang L (2014). Prevalence and associated factors of emotional and behavioural problems in Chinese school adolescents: a cross-sectional survey. Child Care Health Dev.

[23] Sun L, Guo X, Zhang J, Liu HH, Xu SJ, Xu YY (2016). Gender specific associations between early puberty and behavioral and emotional characteristics in children. Zhonghua Liu Xing Bing Xue Za Zhi..

[24] Huang Y, Yan Q, Li Y, Liu J, Yao H, Yan Y (2012). Strengths and Difficulties Questionnaire in 737 primary and middle school students aged 6–17 in Changsha. Zhong Nan Da Xue Xue Bao Yi Xue Ban.

[25] Hu XM, Luan XY, Rou ZWG, Gai GQ (2015). Investigations of psychological status in middle school students in Shihezi city. Lin Chuang Shen Xin Za Zhi.

[26] Chen Q, Ding K, Lu HC, Shi BB, Zhang WW. A cross-sectional study on psychological and behavioral problems of primary and secondary school students. Zheiiang Prev Med. 2013;25(10):30–3. https://d.wanfangdata.com.cn/periodical/zjyfyx201310007 **(in Chinese)**.

[27] Wang XJ, Chen ZM. Investigation of 155 children aged 10–15 using SDQ parent version. Xian Dai Shi Yong Yi Xue. 2006;18(9):662–5. https://d.wanfangdata.com.cn/periodical/nbyx200609031 **(in Chinese)**.

[28] Gao X, Liang J, Wang SF, Zai Y, Lu YB, Shi WH, et al. Analysis on pupils’ current mental health situation and their guardians’ attitude in eight provinces in China. Zhong Hua Ji Bing Kong Zhi Za Zhi. 2013;17(7):592–5. https://d.wanfangdata.com.cn/periodical/jbkzzz201307013 **(in Chinese)**.

[29] WANG JN, Chang Y, Fu JL, Wang L. Relationship between mental health and personality characteristics among junior and senior school students in Shenyang. Zhong Guo Gong Gong Wei Sheng. 2010;26(11):1424–5. https://d.wanfangdata.com.cn/periodical/zgggws201011041 **(in Chinese)**.

[30] Lu AJ, Wang YQ, Wang YF, Zhao ZM. Investigation about emotional and behavioral problems in students aged 7 to 16 years old in Songjiang Shanghai. Jing Shen Yi Xue Za Zhi. 2015;28(2):99–101. doi: 10.1007/BF00165590. https://d.wanfangdata.com.cn/periodical/sdjsyx201502009 **(in Chinese)**.

[31] Yin L, Huang Y, Li Y, Shitu MJ, Xiang Y, Luo Y (2012). Epidemiological investigation on emotion difficulties among elementary and middle school students in Chengdu.

[32] Xiaoli Y, Chao J, Wen P, Wenming X, Fang L, Ning L (2014). Prevalence of psychiatric disorders among children and adolescents in northeast China. PLoS ONE.

[33] Du Y, Kou J, Coghill D (2008). The validity, reliability and normative scores of the parent, teacher and self report versions of the Strengths and Difficulties Questionnaire in China. Child Adol Psych Men.

[34] SDQ: Information for researchers and professionals about the Strengths & Difficulties Questionnaires 2008. https://www.sdqinfo.com. Accessed 04 Feb 2020. 10.1111/j.1475-3588.2007.00443_2.x 32811128

[35] Goodman R (1997). The strengths and difficulties questionnaire: a research note. J Child Psychol Psyc.

[36] Nguyen N, Whittlesey S, Scimeca K, Digiacomo D, Bui B (1994). Parent-child agreement in prepubertal depression: findings with a modified assessment method. J Am Acad Child Psy.

[37] Piacentini JC, Cohen P, Cohen J (1992). Combining discrepant diagnostic information from multiple sources: are complex algorithms better than simple ones?. J Abnorm Child Psychol.

[38] Kuhn C, Aebi M, Jakobsen H, Banaschewski T, Poustka L, Grimmer Y (2017). Effective mental health screening in adolescents: should we collect data from youth, parents or both?. Child Psychiat Hum D.

